# Research trends and hotspot evolution of exercise-regulated myokines: a bibliometric analysis from 2003 to 2023

**DOI:** 10.3389/fphys.2024.1410068

**Published:** 2024-08-01

**Authors:** Zhiyuan Sun, Zekai Wu, Lei Zhu, Xinhe Li, Dongdong Xu, Xuewen Tian, Dewei Mao

**Affiliations:** ^1^ College of Sports Science, Qufu Normal University, Qufu, Shandong, China; ^2^ Institute of Sports Science, Shandong Sport University, Jinan, Shandong, China; ^3^ Graduate Education College, Shandong Sport University, Jinan, Shandong, China

**Keywords:** myokines, exercise, skeletal muscle, irisin, oxidative stress, physical in activity, bibliometric analysis

## Abstract

**Background:**

The lack of physical activity is a common issue in modern society and is considered a major risk factor for various chronic non-communicable diseases. Bioactive factors secreted by skeletal muscle during exercise play a crucial role in inter-organ interactions. Since the concept of “myokines” was proposed in 2004, hundreds of regulatory myokines have been identified. Visual analysis of research on exercise-regulated myokines is significant to explore research hotspots and frontiers in this field.

**Methods:**

Research literature on exercise-regulated myokines from 2003 to 2023 in the “Web of Science” database was used as the data source. Knowledge maps were drawn using “VOS Viewer, CiteSpace, and R-bibliometrix” software.

**Results:**

A total of 1,405 papers were included, showing a fluctuating yet slow growth in annual publications. The United States and China led in the number of publications and collaboration networks. Harvard University ranked first with 120 publications. CIBER (centrality 0.16) and the University of California System (centrality 0.16) were pivotal in advancing this field. PEDERSEN BK led author rankings with 41 publications and 1,952 citations. FRONTIERS IN PHYSIOLOGY ranked first among journals with 64 publications and the highest g-index (39), while PLoS One had the highest h-index (25) and most citations (2,599). Key co-cited reference clusters included #1 skeletal muscle dysfunction, #2 obesity, #6 ASCs, and #7 adaptive immunocytes. Pontus Boström’s paper had a notable citation burst intensity of 77.37. High-frequency keywords were “exercise” (509), “skeletal muscle” (452), and “expression” (293), with long-term keywords such as #0 irisin, #2 insulin resistance, #3 transcription, and #6 physical activity. Recently, keywords like “physical exercise,” “resistance exercise,” “aerobic exercise,” “insulin,” and “oxidative stress” have emerged.

**Conclusion:**

Research in the field of exercise-regulated myokines shows an overall upward trend. The focus areas include myokines mediated by different types of exercise, the interaction of irisin-mediated muscle with other organs, and the important role of myokine-mediated oxidative stress in exercise simulation.

## 1 Introduction

Lack of physical activity is a widespread issue in modern society (Katzmarzyk et al., 2022). As early as 2002, the World Health Organization identified it as one of the leading causes of mortality in developed countries (Carmen et al., 2019). The persistent high incidence of non-infectious chronic diseases (NCDs) poses a significant health challenge today. Physical inactivity is considered a common risk factor for many NCDs (Beghi et al., 2019), and increasing physical activity is an effective non-pharmacological intervention for NCDs (Smith, 2018). Besides actively developing new-generation drugs for various chronic non-communicable diseases, exploring non-pharmacological strategies such as lifestyle-based early interventions offers better health benefits and higher safety. Exercise, as a vital proactive health measure, has beneficial effects on all organs in the body. The health-promoting mechanisms of exercise largely depend on the release of exercise-induced factors, with skeletal muscle playing a crucial role (Beghi et al., 2019). The scientific community has long been interested in understanding how local muscle contractions during exercise can induce systemic health effects, how skeletal muscle communicates with other organs, how all organs/tissues/cells in the body respond to exercise, and how these responses translate into health benefits. Traditional views suggest that the neuro-endocrine-immune network plays an essential role in maintaining metabolic homeostasis through integrated regulation during physical exercise. However, this perspective fails to explain why exercise organs (skeletal muscles) can regulate various systemic organs and how inter-organ influences occur. Research over the past 2 decades has shown that the interactions (crosstalk) between organs and tissues during exercise are primarily mediated by various bioactive factors secreted by tissues such as skeletal muscle. Hundreds of myokines have been identified and confirmed to play regulatory roles. However, the physiological functions of most myokines, including how they are secreted and released from muscle and how they exert regulatory effects, remain unclear. For example, besides abV5, are there other potential receptors for irisin? Acute exercise can promote irisin secretion, but the effects of chronic exercise on irisin secretion remain under debate (Bao et al., 2022). Can irisin only be generated by FNDC-5 cleavage? What factors are involved in the regulation of FNDC-5 cleavage?

The concept of “myokines” was formally introduced in 2004, marking its 20th anniversary (Pedersen et al., 2004). The regulation of myokines by exercise is a widely researched topic, and the vast amount of literature makes it challenging to identify research priorities and frontiers. Therefore, a comprehensive retrospective analysis is crucial for understanding the current status, research hotspots, and future development trends of exercise-regulated myokines. Bibliometric analysis is an emerging interdisciplinary analytical method based on statistics and mathematics (Roldan-Valadez et al., 2019). As an effective method for predicting research evolution trends, bibliometrics quantitatively describes, evaluates, and monitors data, providing reasonable and objective results while avoiding researcher bias. In most cases, bibliometric analysis mainly includes two parts: performance analysis and scientific mapping. Performance analysis aims to reveal the contributions of research based on information from regions, disciplines, journals, and authors in publications. Scientific mapping attempts to elucidate the connections between themes in a research field, thereby revealing the knowledge structure and development process.

This study provides a bibliometric analysis based on the Web of Science database. To make the analysis more comprehensive and thorough, 1,405 publications from the past 20 years were collected. The specific objectives are as follows: 1) Analyze the spatial and temporal distribution of research on exercise-regulated myokines and the contributions of journals and authors. 2) Reveal the thematic network and knowledge framework of research on exercise-regulated myokines over the past 20 years. 3) Illustrate the evolution of research hotspots and trends in the field of exercise-regulated myokines. The results of this study are expected to provide guidance for research on exercise-regulated myokines from the perspectives of research focus and hotspot evolution.

## 2 Materials and methods

### 2.1 Data source and search strategy

**FIGURE 1:**
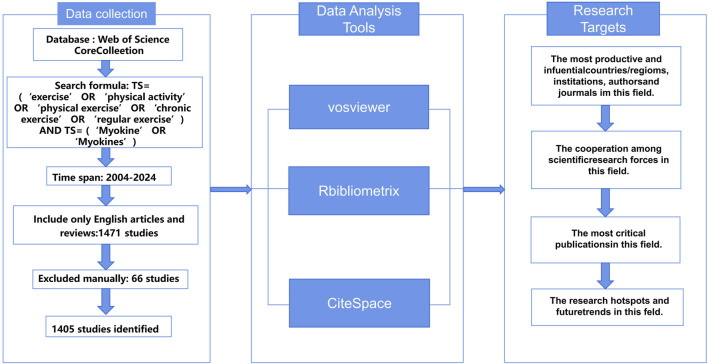
Flow diagram of study selection procedure.

During the collection and screening of literature, we used the Web of Science database as the primary information source and implemented a comprehensive search strategy (Figure 1). To ensure the timeliness of the data and minimize potential bias due to database updates, all searches were independently completed by investigators WZK and SZY on 17 January 2024. We established a specific search strategy, including keyword combinations: (“exercise” OR “physical activity” OR “physical exercise” OR “chronic exercise” OR “regular exercise”) AND (“Myokine” OR “Myokines”), to ensure broad coverage of research related to exercise and myokines. The study period was set from 2003 to 17 January 2024. After the initial screening, we carefully reviewed the titles, abstracts, and full texts to exclude literature unrelated to the research topic. Through this rigorous screening process, we identified 65 records that potentially had issues and ultimately selected 1,405 relevant publications from the initial search results, including 1,010 research articles and 395 review articles.

### 2.2 Data analysis and visualization

We used VOS Viewer 1.6.20 software (Eck and Waltman, 2010) to construct author networks, collaboration networks, and keyword timelines using the “full counting” method. The VOS Viewer software was set to “Create a map based on bibliographic data” under the “Choose type of data” option, and the parameter “Read data from bibliographic database files” was selected based on the source database of the literature. CiteSpace (Chen, 2004) focuses on bibliometric analysis of specific fields to reveal the research structure and development trends of academic disciplines. In this study, CiteSpace was used to analyze co-citation networks, timeline views, citation bursts of references and keywords. It can also create a dual-map overlay of journals, presenting research results in clusters through co-citation networks. The CiteSpace 6.2.R6 software was set with “Time slicing” from 01/01/2023 to 17/01/2024, “Years Per slicing” set to 1 year; “Selection Criteria” set to TOPN with a threshold of 50. Due to the large volume of literature, network pruning was applied to highlight key points, with parameters set to Pathfinder, Pruning sliced networks, and Pruning the merged network. R-bibliometrix (Aria and Cuccurullo, 2017) was used for descriptive analysis to identify leading research countries and journals. The h-index, an indicator of a researcher’s academic achievement, reflects higher academic impact with higher values (Garfield et al., 2006). Additionally, the g-index, based on the h-index, further quantifies a scholar’s influence and academic accomplishments.

### 2.3 Key observational indicators

1) Annual Publication Volume and Citation Frequency: This analysis examines the number of publications and their citation frequencies over the years to understand the growth and impact of research in the field.
2) Author Co-occurrence Relationships and Clustering Characteristics: This involves analyzing the co-occurrence of authors to identify collaboration patterns and clustering characteristics within the research community.
3) Institutional/National Publication Volume and Co-occurrence Relationships: This explores the volume of publications from different institutions and countries, as well as their co-occurrence relationships, to identify key contributors and collaboration networks.
4) Journal Distribution to Understand Citation and Reference Connections: This analysis investigates the distribution of journals to understand how they cite each other and how they are referenced within the field, revealing influential journals and publication trends.
5) Keyword Co-occurrence and Clustering to Show Research Hotspots Distribution: This involves analyzing the co-occurrence of keywords and their clustering to identify the distribution of research hotspots within the field.
6) Keyword Burstiness to Identify Frontier Hotspots in the Field: This examines the sudden increases in keyword usage to identify emerging trends and frontier hotspots in the research area.
7) Keyword Clustering Timeline to Present the Dynamic Evolution of Research Themes and Hotspots: This presents a timeline of keyword clusters to show the dynamic evolution of research themes and hotspots over time, providing insights into how the field has developed and where it is heading.

## 3 Results

### 3.1 Development trends and national analysis

**FIGURE 2:**
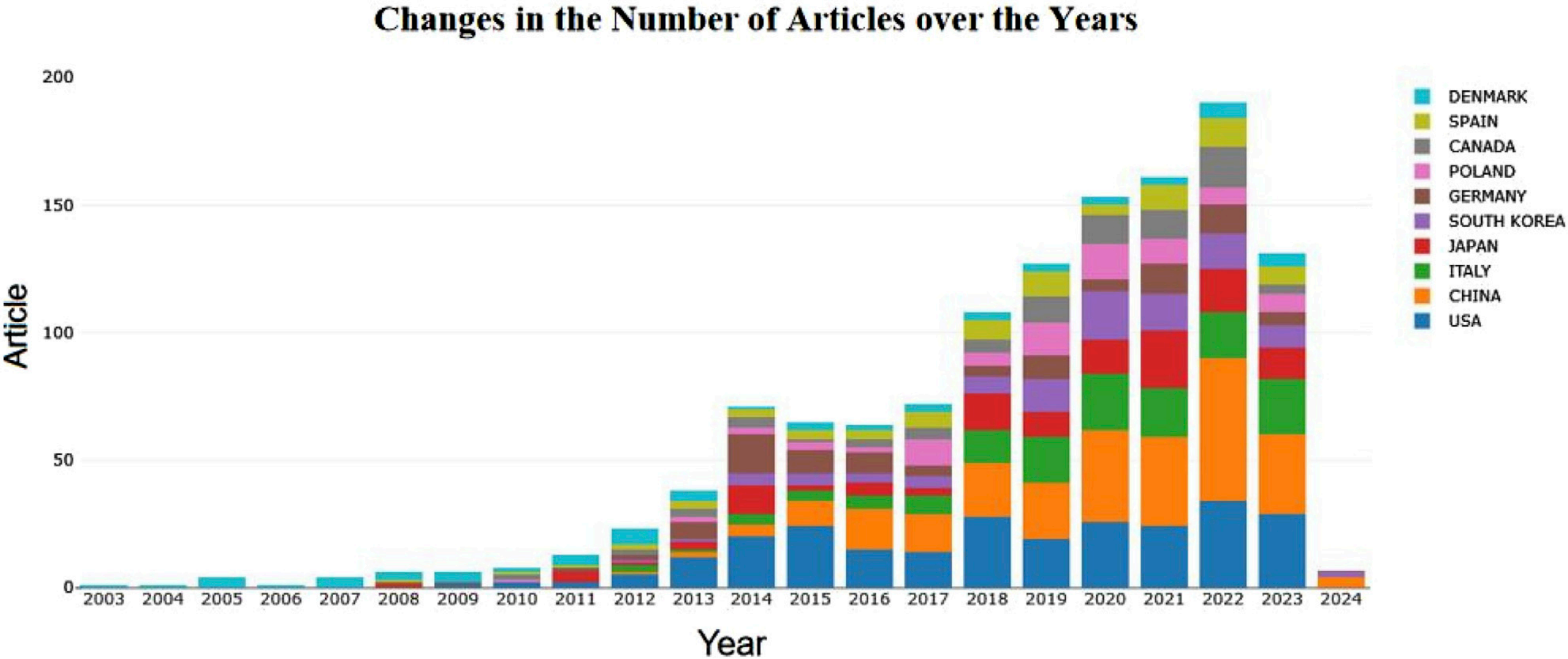
Annual number of documents published.

The number of publications is an important indicator of the research interest and development trends in a particular field. To clearly demonstrate the volume of research on exercise-regulated myokines, we used Excel to statistically organize the literature samples obtained after retrieval and screening, based on the time series from 2003 to 2024. The distribution of publication quantities is shown in Figure 2. From 2003 to 2022, the number of research papers on exercise-regulated myokines has shown a significant upward trend. During this period, especially between 2003 and 2014, the annual number of related publications steadily increased. Although there was a brief decline in 2015 and 2016, the publication volume quickly rebounded between 2017 and 2022, reaching a peak of 223 papers in 2022. However, the number of publications in 2023 unexpectedly dropped to 159 papers, but the overall trend still indicates growth.

**FIGURE 3:**
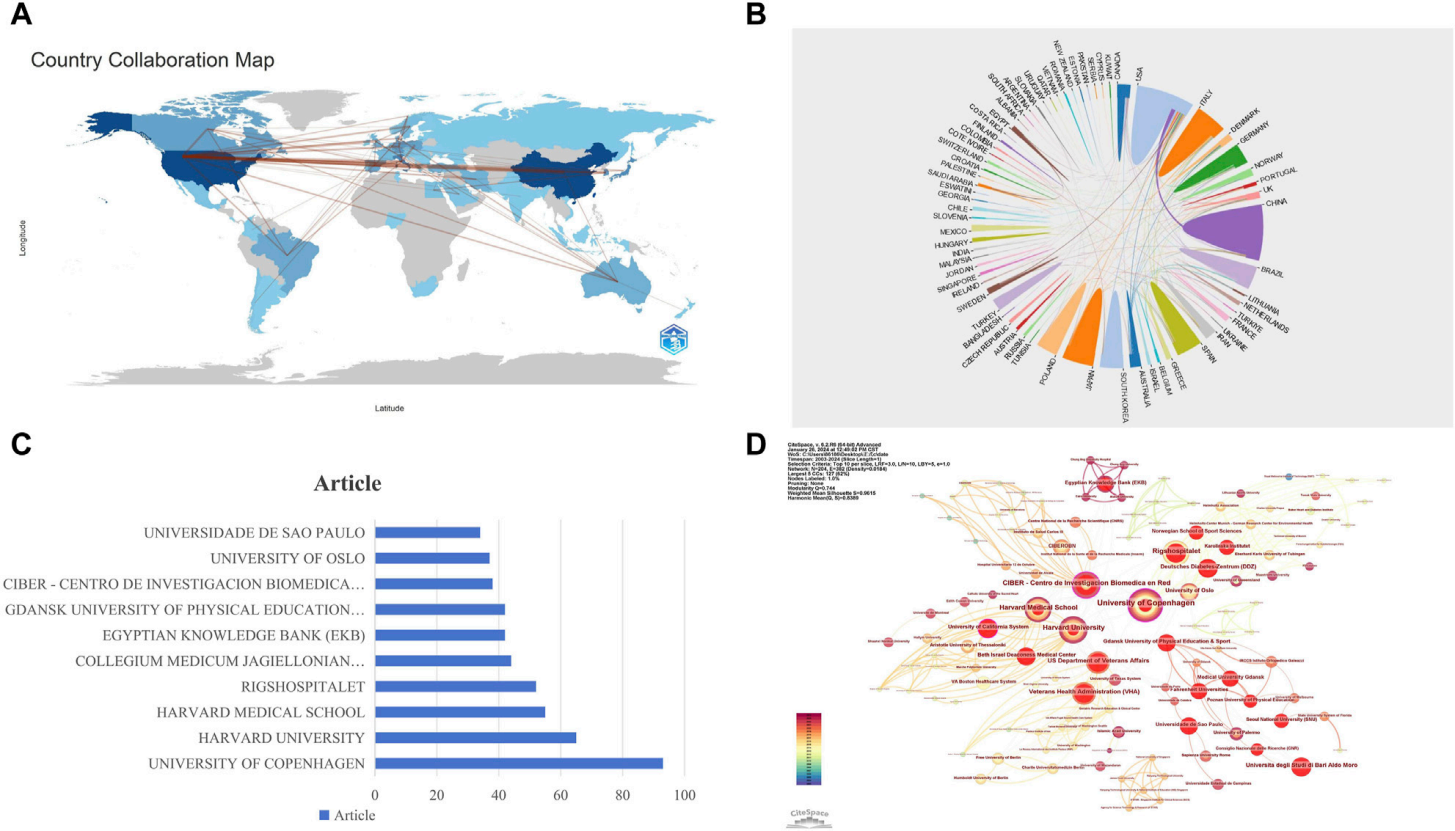
Visualization of National and Institutional Collaboration Networks. **(A)** World Network of National Collaboration Relationships: lines between countries on the map represent their collaborative links, with the thickness of each line indicating the closeness of the collaboration between the two countries. **(B)** Geographical Distribution of International Collaboration Networks. **(C)** Publication Volume of the Top Ten Institutions. **(D)** Institutional Collaboration Network: nodes represent institutions (the larger the circle, the higher the number of publications). Lines between nodes indicate collaboration on the same paper (the wider the line, the higher the frequency of collaboration). The size of the circles represents the level of collaborative activity of the institutions.

The articles involved in this study come from 73 countries worldwide, showcasing the broadness and diversity of international scientific collaboration. The map in Figure 3A visually represents the strength of cooperative relationships between countries, illustrating the intensity of their collaborations. The United States and China stand out in this network, displaying dense cooperation links, which indicates that these two countries play central roles in international scientific collaboration. This close cooperation may stem from their strong research capabilities, abundant resources, and open research environments. Figure 3B reveals the geographical distribution of the international cooperation network, highlighting particularly frequent academic exchanges among European countries. This may be related to geographical proximity and shared research cultures. In contrast, cooperation among African countries is relatively sparse, which may require further analysis to explore underlying reasons, such as resource allocation, research infrastructure, or policy support.

### 3.2 Analysis of institutions

In the field of research on exercise-regulated myokines, a total of 1,408 institutions have published papers. According to the data in Figure 3C, the University of Copenhagen ranks first with 93 publications, followed closely by Harvard University and Harvard Medical School with 65 and 55 publications, respectively. To further reveal the collaboration relationships among these institutions, we used CiteSpace to construct an institutional collaboration network graph (Figure 3D), which clearly displays the close connections between different institutions. In the centrality analysis, we found that the University of California System (0.16), CIBER - Centro de Investigacion Biomedica en Red (0.16), and Harvard University (0.15) all have centrality values exceeding 0.15. This metric indicates that these three institutions play crucial roles in advancing this research field. Although University of California System and CIBER - Centro de Investigacion Biomedica en Red are not the most prolific in terms of publication volume, their high centrality value signifies that its research results have significant influence in the academic community and a wide collaboration network. These findings highlight the importance of inter-institutional collaboration in scientific research and the leading positions of certain institutions in specific research areas.

### 3.3 Analysis of authors and co-cited author

**FIGURE 4:**
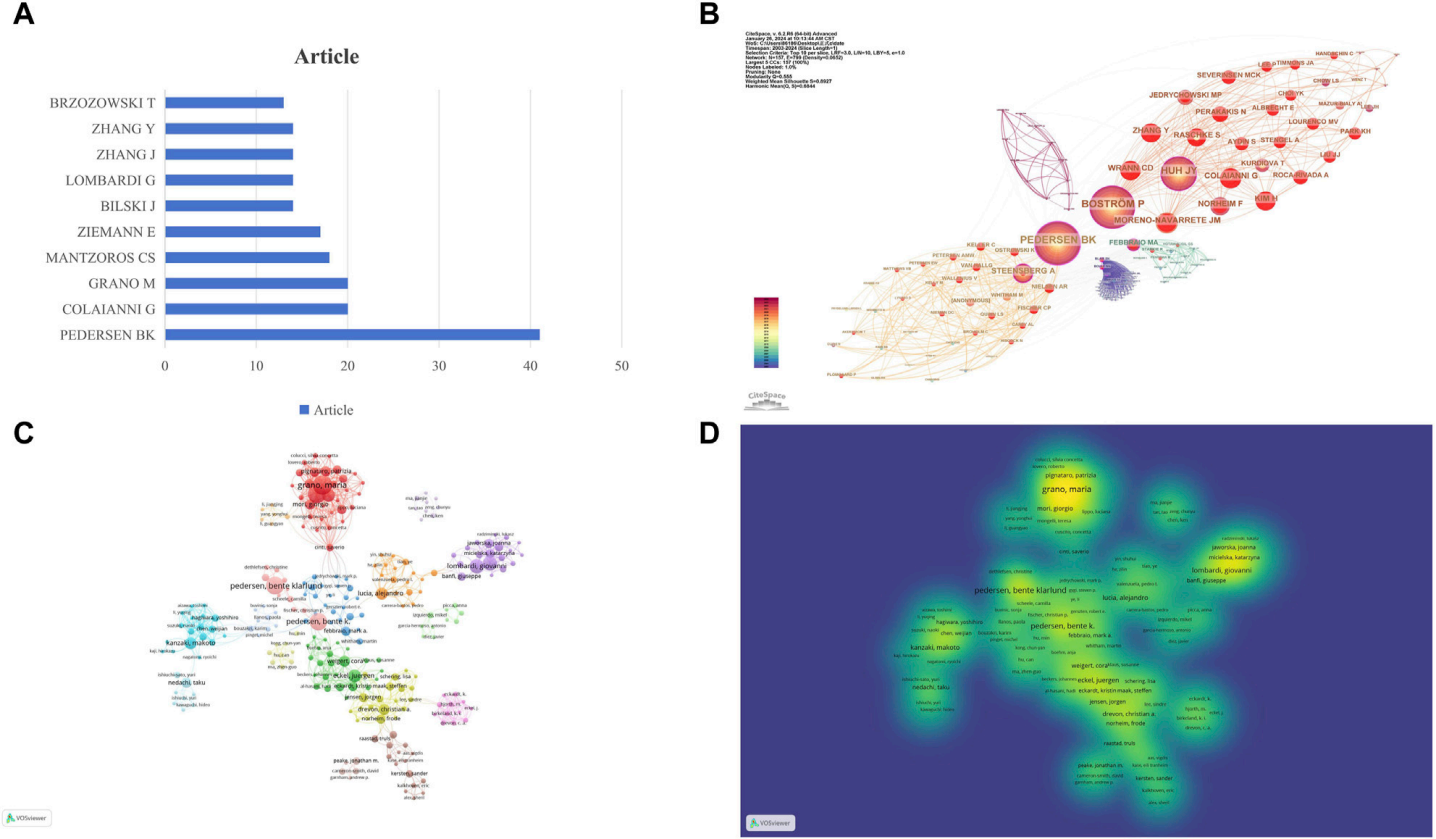
Visualization of Author and Co-cited Author Networks. **(A)** Publication Volume of the Top Ten Authors: this figure shows the number of publications by the top ten authors in the field. **(B)** Co-cited Author Network: each circle represents an author, and the size of the circle is determined by the number of publications (the larger the number, the larger the circle). Authors in the same cluster share the same color. Lines between circles represent co-citations (the stronger the collaboration, the thicker the line). The number of total link strengths reflects the overall co-citation strength between authors. **(C)** Research Author Collaboration Network: each circle represents an author, and the size of the circle is determined by the number of publications (the larger the number, the larger the circle). Authors in the same cluster share the same color. Lines between circles represent co-authorship (the stronger the collaboration, the thicker the line). The number of total link strengths reflects the overall co-authorship strength between authors. **(D)** Research Author Collaboration Density: the color intensity represents the research productivity heat of core experts in the field, with darker colors indicating higher productivity. The collaboration degree between core expert teams is represented by their density distribution, with closer distances indicating stronger collaboration.

In the in-depth analysis of research on exercise-regulated myokines over the past 20 years, we ranked authors based on the number of papers they published to identify prolific researchers in this field. According to the data in Figure 4A, PEDERSEN BK ranks first with 41 published papers, followed by COLAIANNI G and GRANO M, each with 20 papers. In the academic community, the citation frequency of an author’s work is a key indicator of their research impact. By analyzing co-citation data in the references, we constructed a co-citation network of authors (Figure 4B). In this network, PEDERSEN BK ranks first with 1952 co-citations, followed by CINTI S (1019 co-citations) and FEBBRAIO MA (827 co-citations). These data further confirm the significant contributions of these authors to the field of myokine research. To gain a deeper understanding of the collaboration relationships among authors, we used VOS Viewer software to construct author collaboration and density networks, including only authors who have published at least 20 papers (Figures 4C, D). Through the analysis of the author collaboration network, we identified over 10 core teams, each named after a representative author, such as PEDERSEN BK. These team divisions reveal the close collaboration among authors and their crucial roles in advancing the field of myokine research. Overall, these authors and their teams have significant academic influence in this field. Their research outputs are not only numerous but also of high quality, having a profound impact on subsequent research.

### 3.4 Analysis of co-cited journals

**FIGURE 5:**
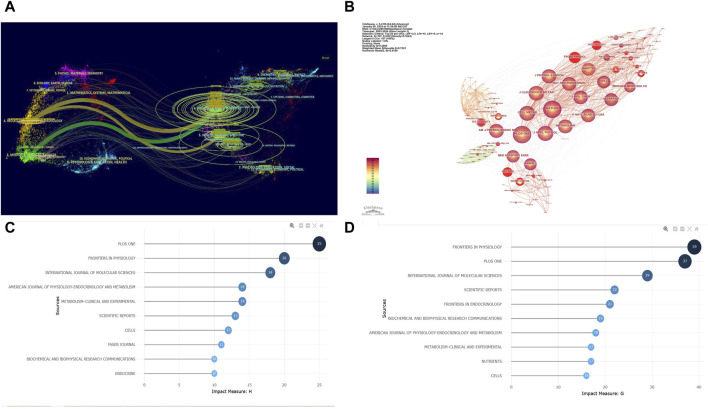
Visualization of Co-cited Journals. **(A)** Double Map Overlay of Journals: The left side represents the basic curve map of citing journals, while the right side represents the basic curve map of cited journals. The wavy lines connect the relationships between current research and foundational research. The numbers inside the ellipses indicate the publication volume of each discipline. Z-score is a statistical measure used to indicate the position of a given data point within a dataset. **(B)** Co-cited Journal Network: each circle represents a journal, with the size of the circle determined by the number of publications (the larger the number, the larger the circle). Journals in the same cluster share the same color. Lines between circles represent co-citations (the stronger the collaboration, the thicker the line). The total link strength reflects the overall co-citation strength between journals. **(C)** h-index of the Top 10 Journals. **(D)** g-index of the Top 10 Journals.

To explore the most popular publishers in the field of exercise-regulated myokines over the past 20 years, we ranked all relevant journals by the number of publications. A double map overlay analysis was used to visualize the citation relationships between journals and reveal cross-disciplinary intersections. Figure 5A shows a double map overlay of journals to illustrate the disciplinary distribution of journals based on studies of exercise-regulated myokines. Citation relationships are indicated by colored paths between the citing and cited journals. A two-color primary citation pathway is identified by the mapping, meaning that research published in journals in the field of molecular/biology/genetics and medicine/medical/clinical were primarily cited by research published in molecular/biology/immunology, medical/medical/clinical, health/nursing/medicine journals. In terms of publication volume, FRONTIERS IN PHYSIOLOGY ranks first with 64 papers, followed by the INTERNATIONAL JOURNAL OF MOLECULAR SCIENCES (54 papers) and PLoS One (37 papers). Figure 5B further depicts the co-cited journal visualization network, where circles represent journals and lines indicate co-citation relationships between journals. Analysis shows that PLoS One is the most frequently co-cited journal (2,599 times), followed by the JOURNAL OF APPLIED PHYSIOLOGY (2,290 times) and CELL METABOLISM (1,847 times). Figures 5C, D display the h-index and g-index of the top 10 journals, showing that PLoS One has a high h-index (25) and FRONTIERS IN PHYSIOLOGY has a high g-index (39). These metrics indicate that PLoS One and FRONTIERS IN PHYSIOLOGY have significant academic influence and publication potential in the field of exercise-regulated myokines, making them recommended publishing platforms for researchers in this area.

### 3.5 Co-cited reference analysis

**FIGURE 6:**
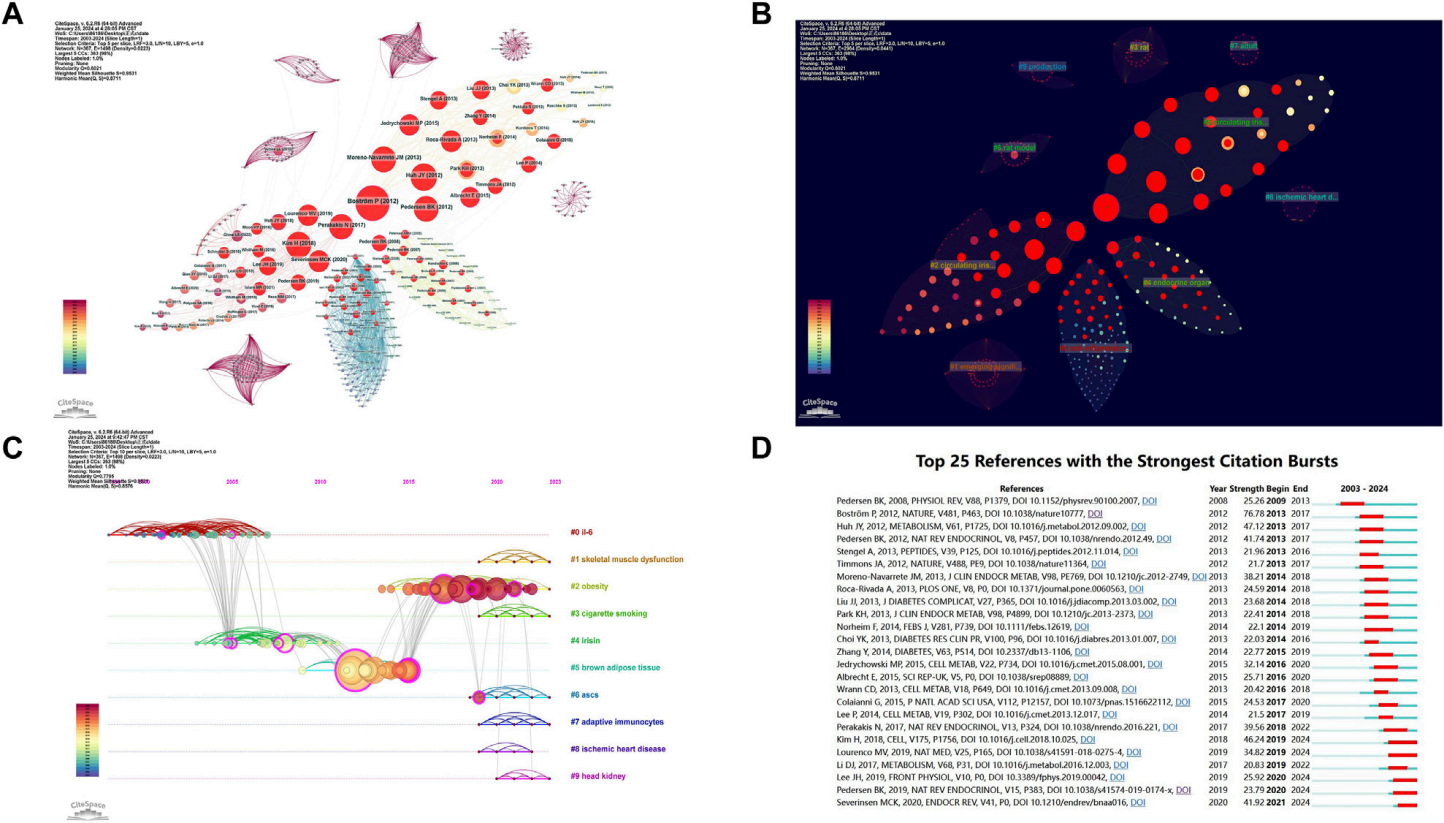
Visualization of Co-cited References. **(A)** Co-cited Reference Network: nodes represent co-cited references, with red circles indicating references experiencing citation bursts. **(B)** Co-cited Reference Clusters: a total of 10 clusters were identified in the network graph. **(C)** Timeline of Cluster Analysis: this timeline shows the temporal evolution of the identified clusters. **(D)** Top 25 Most Cited References: this displays the 25 references with the highest citation counts.

Using CiteSpace software for an in-depth co-citation analysis, we revealed important literature and their interconnections within this field. This analysis constructed a complex network comprising 367 nodes and 1,498 links (Figure 6A). Each node represents a widely cited paper, and the size of the node reflects the total citation frequency of that paper. Through this visualization tool, we can intuitively observe the connections between papers and the knowledge structure within the field. Based on the co-occurrence network, we used the log-likelihood ratio (LLR) algorithm to perform a cluster analysis of the keywords in the literature, dividing the data into 10 main clusters (Figure 6B). These clusters reflect the breadth and diversity of the research field.

The reasonableness and validity of the clustering results were determined by the modularity value (Q value) and silhouette value (S value): Q value > 0.3 indicates a significant clustering structure, S value > 0.5 indicates reasonable clustering results, and S value > 0.7 indicates highly reliable clustering results. The results showed: Q value = 0.7795 > 0.3, S value = 0.9531 > 0.7, indicating that the keyword clustering results are highly significant and reasonable. The smaller the cluster number, the higher the research intensity of that cluster. This allows us to gain insights into the dynamic changes in research hotspots. Notably, the clusters #1 skeletal muscle dysfunction, #2 obesity, #6 ASCs, and #7 adaptive immunocytes have been particularly emphasized in recent years by the academic community (Figure 6C).

By analyzing citation bursts, we can precisely identify articles that have garnered significant attention from scholars in the same field and filter out those with a substantial impact on future research. Figure 6D shows the top 25 most frequently cited articles. Among these, the study by Pedersen BK published in 2009 in “Physiological Reviews” (V88, P1379) began to gain attention early on (Pedersen and Febbraio, 2008). Notable recent citation bursts include Pedersen BK’s 2019 study in “Nature Reviews Endocrinology” (V15, P383) (Pedersen, 2019), Lee JH’s 2019 study in “Frontiers in Physiology” (V10, PO) (Lee and Jun, 2019), and the 2020 study by Severinsen MCK and Pedersen BK in “Endocrine Reviews” (V41, P1) (Krogh and Klarlund, 2020). Of particular note is Pontus Boström’s article published in 2012 in “Nature” (V481, P463) titled “A PGC1-α-dependent myokine that drives brown-fat-like development of white fat and thermogenesis” (Boström et al., 2012). This article has a citation burst intensity of 77.37, the highest among all the articles, highlighting its significant academic influence and importance. This study not only revealed the link between exercise and myokines but also explored the potential mechanisms and related risk factors of exercise-induced myokines, profoundly impacting subsequent research.

### 3.6 Analysis of co-occurrence keywords

**FIGURE 7:**
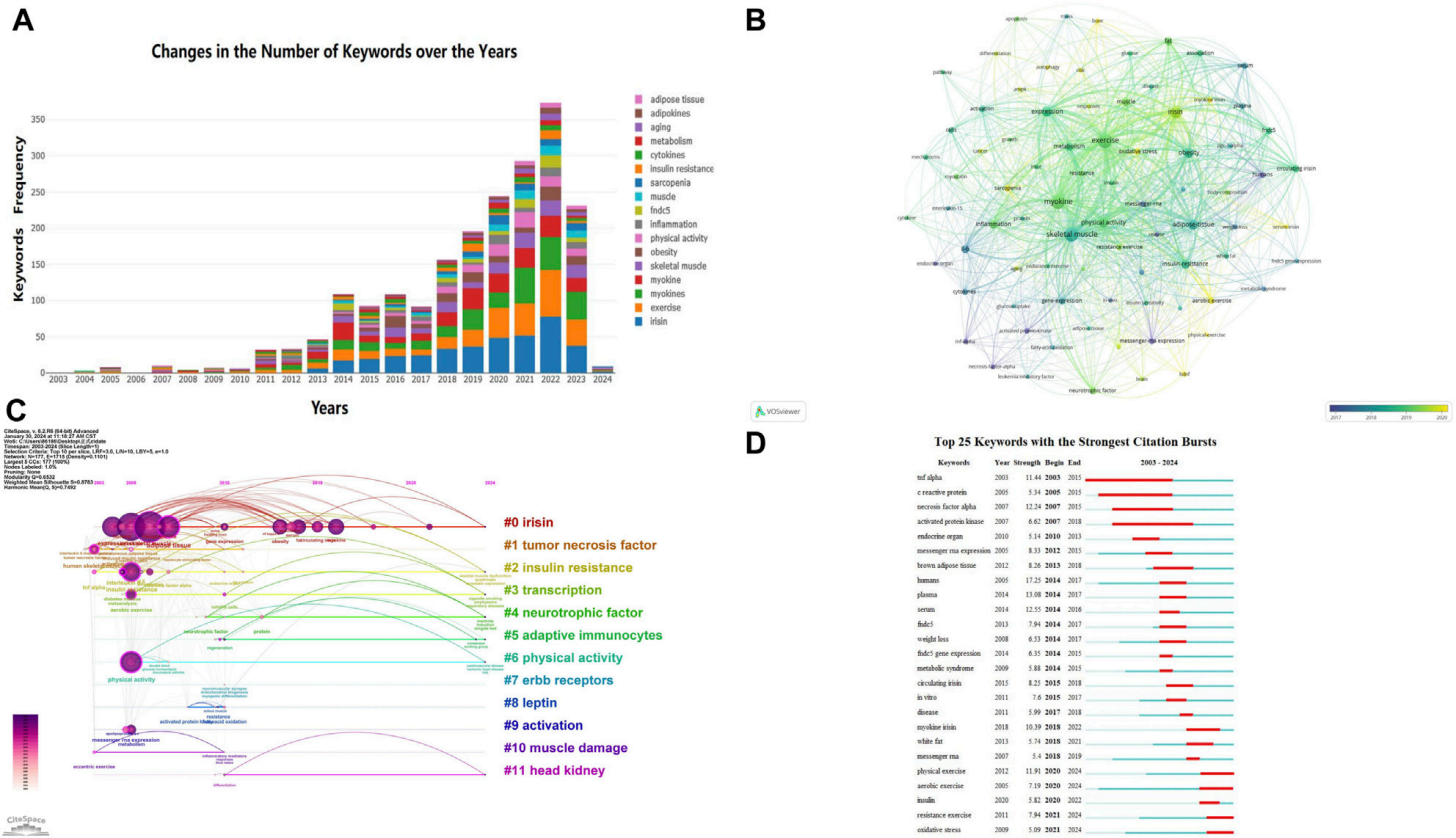
Visualization of Keywords. **(A)** Annual Changes in the Top 20 Keywords by Research Frequency: this figure shows how the top 20 most frequently researched keywords have changed over the years. **(B)** Trends in Keyword Frequency Over Time: in the overlay visualization map, different colors are assigned to keywords based on their average occurrence time. From the timeline perspective, blue keywords appeared earlier than yellow keywords. **(C)** Trends in Keyword Frequency Over Time: similar to panel B, this overlay visualization map assigns different colors to keywords based on their average occurrence time. Blue keywords appeared earlier than yellow keywords. **(D)** Visualization of the Top 25 Keywords with the Most Citation Bursts in CiteSpace: this figure shows the top 25 keywords that experienced the most significant citation bursts.

Keywords reflect the themes of articles and can be used to analyze the key points and research frontiers in a specific field. Figure 7A mainly displays the top 20 most frequently studied keywords, with “exercise” (509 times), “skeletal muscle” (452 times), and “expression” (293 times) occupying the top three positions. Notably, over the past 3 years, research on “irisin” has been particularly prominent. Using VOS Viewer software for keyword analysis, we identified 81 keywords that appeared more than 30 times. The average publication year was used to determine the color of each keyword. By referring to the corresponding timeline, we can see that terms such as “oxidative stress,” “sarcopenia,” “risk,” “bone,” “aerobic exercise,” “physical exercise,” “BDNF,” “autophagy,” “myokine irisin,” and “serum irisin” have emerged as recent research hotspots (Figure 7B).

Using CiteSpace software, we generated a keyword timeline map (Figure 7C) to understand the temporal distribution of hotspot terms in this field and predict future development trends. In the figure, straight lines represent 0–11 different clusters, with circular nodes on the lines corresponding to keywords under these clusters. The larger the node, the higher the frequency of the keyword. Among them, #0 irisin, #2 insulin resistance, #3 transcription, and #6 physical activity have long time spans, indicating sustained research interest. Figure 7D shows the top 25 keywords with citation bursts. The blue line represents the time interval, and the red line represents the period of keyword bursts (Chen et al., 2014). The analysis of keyword bursts further reveals the evolution of research trends. Keywords such as “tnf alpha,” “c reactive protein,” “necrosis factor alpha,” and “activated protein kinase” have been of interest since 2003, whereas keywords like “physical exercise,” “resistance exercise,” “aerobic exercise,” “insulin,” and “oxidative stress” have become recent research hotspots. This indicates that these research themes have gained considerable attention recently and may become new research hotspots in the coming years. The keyword “aerobic exercise” had the strongest citation burst in 2021, with a long burst duration, highlighting the importance of aerobic exercise in regulating myokines.

## 4 Discussion

Exercise can reduce the risk of a range of diseases. The awareness that exercise can regulate changes in organs and tissues dates back more than 50 years. Goldstein first proposed that contracting skeletal muscle cells could release humoral factors that regulate metabolism (Goldstein, 1961). In paralyzed patients without efferent or afferent nerve impulses, electrical stimulation of paralyzed muscles induces the same type of physiological changes as in healthy individuals (Mohr et al., 1997). Clearly, the signaling pathway from exercising skeletal muscle to other organs is not solely mediated by the nervous system. Since then, researchers have realized that during exercise, muscles might interact with other organs and tissues by releasing “humoral factors” into the bloodstream, thereby exerting regulatory effects. In 2003, PEDERSEN BK discovered that skeletal muscle produces and releases interleukin-6 (IL-6) into the circulation (Pedersen et al., 2003). Subsequent studies showed that IL-6 has various metabolic effects in other parts of the body, establishing IL-6 as an exercise factor. Considering the numerous physiological, metabolic, and immune effects of exercise, it is evident that more than one exercise factor might be identified. In 2004, PEDERSEN BK introduced the term “myokines” and proposed that “cytokines and other peptides that are produced, expressed, and released by muscle fibers and exert autocrine, paracrine, or endocrine effects should be classified as myokines” (Pedersen et al., 2004). During exercise, skeletal muscle can act as a secretory organ releasing myokines to regulate muscle metabolism in an autocrine manner and also regulate the function of other tissues and organs through paracrine/endocrine mechanisms, though the underlying molecular mechanisms remain unclear (Krogh and Klarlund, 2020).

As researchers focus on the role of exercise in human health and disease development, exercise-induced cytokines have emerged as a new mechanism for regulating metabolic homeostasis, becoming a hot topic in contemporary sports science and sports medicine research. Currently, researchers have identified hundreds of myokines (Graf and Ferrari, 2019; Krogh and Klarlund, 2020) and are beginning to examine the role of myokines in the crosstalk between muscle and other organs, including adipose tissue, bone, liver, intestines, pancreas, blood vessels, brain, and skin (Pourteymour et al., 2017; Laurens et al., 2020). This study uses bibliometric and visual analysis, employing CiteSpace, VOS Viewer, and R-bibliometrix software to analyze and summarize 1,405 papers in the field of exercise-regulated myokines from the WOS database. The corresponding visual maps illustrate the research overview and hotspot trends in the field of exercise-regulated myokines over the past 20 years.

### 4.1 Spatial and temporal distribution, contributions from journals and authors

A total of 1,405 papers were included in this study, covering research on exercise-regulated myokines from 2003 to 2023. The volume of publications in this field has been rising year by year, attracting increasing attention from international researchers, and overall showing a trend of growth and development. Among the various countries and regions, China and the United States have the highest number of publications, followed by Italy, Japan, Spain, South Korea, and Poland, indicating that research outcomes in this field are primarily concentrated in developed countries in Europe and North America. When analyzing publication volumes at the national level, we noticed a decline in contributions from countries such as China in 2023. This downward trend may be related to delays in updating articles in the database. Additionally, the COVID-19 pandemic may have restricted the quantity and quality of experimental research, thereby affecting the output of papers. This observation suggests that global health crises have profound impacts on scientific research activities, particularly in the biomedical field, which relies heavily on experimental support.

The top three institutions with the highest number of publications are the University of Copenhagen, Harvard University, and Harvard Medical School, corresponding to the publication volumes at the national level. It is worth noting that Harvard University and Harvard Medical School were counted separately; in reality, Harvard University has the highest number of publications in this research area. Harvard University focuses on myokines and their physiological effects, particularly how myokines influence insulin secretion, energy metabolism, and muscle-brain communication. They have made significant contributions to understanding how these proteins help regulate metabolic processes and affect diseases such as obesity and type 2 diabetes. According to the search results, Harvard University’s research expenditure is quite substantial, with an annual average R&D expenditure of $1.01 billion, providing a solid foundation for its research activities. In contrast, although the University of California System and CIBER - Centro de Investigacion Biomedica en Red do not have as many publications, they both exhibit the highest centrality (0.16). This indicates that these two institutions play crucial roles in advancing academic progress, influencing research directions, and collaborating with other academic units in the field. The University of California, Berkeley, within the University of California System, has conducted extensive research on the regulation of myokines, focusing on the role of these muscle-secreted proteins in metabolic health and disease. For example, research has explored how myokines affect bone metabolism, energy balance, and metabolic diseases such as diabetes. CIBER - Centro de Investigacion Biomedica en Red is a Spanish biomedical research network that collaborates with institutions worldwide, including those within the University of California System. Their research typically focuses on the endocrine function of skeletal muscle and the systemic effects of myokines, including their roles in metabolic regulation and chronic diseases. Collaboration between these institutions often occurs through joint research projects, conferences, and publications. These partnerships facilitate a more comprehensive and impactful understanding of myokine biology by pooling resources and expertise. Such cooperation is essential for advancing the field and developing potential treatment strategies for metabolic diseases.

The most active authors in this field are PEDERSEN BK, COLAIANNI G, GRANO M, MANTZOROS CS, and ZIEMANN E. PEDERSEN BK’s research revealed that muscles are not just organs of movement but also possess endocrine functions, capable of secreting various factors beneficial to other parts of the body. This discovery suggests that regular exercise can regulate health benefits through these muscle factors, particularly in reducing chronic inflammation (Febbraio and Pedersen, 2020). Her research also emphasizes the role of exercise in regulating immune function and reducing systemic inflammation, which is especially important for patients with rheumatoid arthritis and other inflammatory diseases (Benatti and Pedersen, 2015). Additionally, Pedersen’s research involves the role of muscle factors in regulating glucose homeostasis and metabolic health. For example, she found that interleukin-6 (IL-6) plays a crucial role during muscle contraction, impacting the management and prevention of metabolic diseases such as type 2 diabetes (Pedersen, 2010).

Maria Grano and Graziana Colaianni have established a close collaborative relationship in the field of myokine research, particularly focusing on the role of irisin. Their collaboration has significantly advanced our understanding of how irisin affects skeletal and muscular health. Their research indicates that the myokine irisin plays a crucial role in bone and muscle health. They found that irisin can significantly enhance cortical bone mineral density and strength, as well as promote osteoblast differentiation and bone formation (Colaianni et al., 2015). Additionally, irisin plays a key role in the interaction between muscle and bone by promoting bone formation and inhibiting bone resorption, thus enhancing bone health (Colaianni et al., 2016). These studies reveal multiple mechanisms by which irisin maintains bone health, emphasizing its potential as a therapeutic target.

Christos S. Mantzoros has also made significant contributions to irisin research. He has studied the effects of different types of exercise on irisin levels in individuals with and without metabolic syndrome, revealing the importance of irisin in regulating energy metabolism (Huh et al., 2015). He also explored the physiological role of irisin in regulating glucose homeostasis, uncovering its impact on energy metabolism and insulin sensitivity (Perakakis et al., 2017). Another study by Christos S. Mantzoros confirmed the presence of irisin as a circulating hormone and its role in regulating metabolism and adipose tissue function, further solidifying irisin’s status as a crucial myokine (Polyzos et al., 2015).

E. Ziemann has focused on the role of myokines in muscle and metabolic functions. His research examined how high-intensity circuit training affects the levels of various myokines, including irisin, interleukin-6, and brain-derived neurotrophic factor (BDNF), and their effects on cognitive function and metabolic health (Gmiat et al., 2017). Another study investigated the impact of whole-body cryotherapy on irisin levels, demonstrating that this therapy can enhance muscle performance and metabolic outcomes (Dulian et al., 2015).

The journals with the highest number of publications and citations in this field are predominantly hosted by European and American countries, indicating that authors and journals from these regions have greater influence and credibility. PLoS One and FRONTIERS IN PHYSIOLOGY have high publication volumes and high h-index and g-index scores. Researchers in this field should closely monitor these journals to stay updated on the latest research developments. Currently, the focus in the field of exercise-regulated myokines is on the molecular mechanisms by which different types of exercise mediate myokines and the interaction between muscles and other organs. The research trends and hotspots in this field include “physical exercise,” “resistance exercise,” “aerobic exercise,” “insulin,” and “oxidative stress.”

### 4.2 Knowledge framework and research trends

#### 4.2.1 Myokines mediated by different types of exercise

In the keyword burst network (Figure 7D), “physical exercise,” “aerobic exercise,” and “resistance exercise” have recently shown significant bursts, with “aerobic exercise” starting to stand out in 2005 and experiencing a major burst since 2021. This suggests that different exercise modalities (aerobic and resistance training) have become recent research hotspots. Existing research confirms that different types of physical activity can influence skeletal muscle metabolism through various pathways, and the secretion of myokines is also affected by the type of physical activity (Mammucari et al., 2007). After aerobic training, muscles release specific myokines such as BAIBA, FGF-2, and Musclin; after resistance training, muscles release specific myokines such as angiopoietin-like 4 (ANGPL-4), BMP-7, and Decorin; other myokines like irisin, IL-15, and FGF-21 are secreted and released after both types of exercise (Barbalho et al., 2020). Besides the type of exercise, some myokines are preferentially secreted by fast muscle fibers, such as Musclin, FGF-21, osteoprotegerin, and angiopoietin (Subbotina et al., 2015; Sabine et al., 2018); irisin and myonectin are mainly secreted by slow muscle fibers (Bostrm et al., 2012), and the secretion characteristics of most other myokines are still unclear. Additionally, the secretion and function of myokines are influenced by exercise intensity, exercise duration (single bout, long-term), and other individual factors such as gender and age. For example, the secretion and function of IL-6 are closely related to the duration and type of exercise (Knaepen et al., 2010).

Currently, the “Molecular Transducers of Physical Activity Consortium” (MoTrPAC) project, established by the National Institutes of Health (NIH) Common Fund, is classifying biomolecules affected by physical activity, mapping a comprehensive profile of exercise factors, and attempting to link changes in exercise factors to the benefits of physical activity. This project aims to determine how exercise factors change with age, gender, body composition, health status, and long-term exercise exposure. Therefore, when exploring the mechanisms by which myokines influence physical activity and non-communicable diseases (NCDs) and when implementing exercise interventions, it is crucial not to overlook the prerequisites for myokine secretion and function. In-depth research on exercise factors will provide theoretical foundations for understanding the mechanisms of exercise-regulated metabolism, scientifically selecting exercise frequency, intensity, duration, and type, and applying exercise therapy in the prevention and treatment of metabolic diseases.

#### 4.2.2 Interaction between muscle and other organs mediated by irisin

Irisin is a myokine that has been widely studied in recent years. Exercise increases the expression and cleavage of FNDC5 (fibronectin type III domain-containing protein 5) through activation of the skeletal muscle PGC-1α signaling pathway. The cleaved FNDC5, secreted from skeletal muscle cells, is irisin (Schumacher et al., 2013). Irisin primarily enhances mitochondrial activity by activating signaling mechanisms such as MAPK and uncoupling protein-1 (UCP-1) in target cells. This leads to various biological effects, including the conversion of white adipose tissue to brown adipose tissue, increased thermogenesis, promotion of energy metabolism, improvement of insulin resistance (IR), involvement in bone metabolism, enhancement of brain function, and improvement of cognitive abilities (Bostrm et al., 2012; Hashemi et al., 2016; Butt et al., 2017; Mahgoub et al., 2018; Tang et al., 2019; Ye et al., 2019; Pullisaar et al., 2020). Multiple studies have shown that irisin can regulate lipid metabolism. Overexpression of FNDC5 in insulin-resistant mice can increase circulating irisin levels, reduce adipose tissue, and decrease body weight, suggesting that irisin can improve insulin sensitivity, increase energy expenditure, and promote the browning of white adipose tissue (WAT) (Bostrm et al., 2012). Additionally, cell experiments have shown that irisin can enhance lipolysis in 3T3-L1 cells, indicating that it is a potential lipolytic factor (Xiong et al., 2015). In bone metabolism, irisin positively affects osteoblast differentiation (Colaianni et al., 2014) and inhibits osteoclast differentiation (David, 2015). Qiao et al. (2016) revealed *in vitro* that irisin can directly act on osteoblasts through the P38/MAPK/ERK signaling pathway, enhancing alkaline phosphatase (ALP) activity and calcium ion deposition, thereby promoting osteoblast proliferation, differentiation, and mineralization. Zhang et al., (2017) found that irisin can inhibit osteoclast differentiation by inhibiting RANKL receptor activity or the NFATc1 pathway. *In vivo* studies have shown that recombinant irisin administration in male mice increases cortical bone mass and bone strength by stimulating bone formation and reducing the number of osteoclasts (Graziana et al., 2015). Numerous epidemiological and clinical data suggest that exercise has positive effects on brain health and cognitive function, though the mechanisms are complex. Aerobic exercise can upregulate brain irisin levels in Alzheimer’s disease mice and reduce anxiety levels (Uysal et al., 2018). Irisin has been shown to be secreted in response to exercise, crossing the blood-brain barrier and increasing brain BDNF levels (Moon et al., 2016). However, there is some debate about whether irisin is involved in the endocrine communication between muscle and brain (Albrecht et al., 2015; Christiane, 2015). Further research is needed to better understand the muscle-brain interactions and the role of myokines in these mechanisms.

The response of irisin to exercise depends on the intensity, duration, type of exercise, and training status (Kraemer et al., 2013; Daskalopoulou et al., 2014; Norheim et al., 2014; Dennis et al., 2015). Long-term exercise can improve the decreased circulating irisin concentrations caused by aging and a sedentary lifestyle. However, it is still unknown whether acute exercise can systematically alter irisin levels to confer health benefits. Additionally, there is significant controversy regarding the effect of exercise on circulating irisin. Some reports support that exercise promotes the entry of irisin into the bloodstream (Huh et al., 2012; Jedrychowski et al., 2015), while other studies suggest that exercise does not affect irisin levels (Kurdiova et al., 2014). Future research should focus on irisin receptors and address questions such as: Can irisin only be generated by FNDC-5 cleavage? What factors are involved in the regulation of FNDC-5 cleavage?

#### 4.2.3 Oxidative stress

Oxidative stress is one of the hottest burst keywords in the field of exercise-regulated myokines from 2021 to 2023, with the highest burst intensity. Oxidative stress refers to the imbalance between the production and clearance of reactive oxygen species (ROS) and reactive nitrogen species (RNS) in the body, leading to oxidative damage and a pathological response in cells and tissues (Sonal and Ekta, 2023). With the development of translational medicine, researchers have been identifying biological targets that promote health through exercise and designing compounds, known as “exercise pills,” to achieve “exercise mimetics.” “Exercise mimetics” are an emerging form of therapy that simulates the beneficial effects of physical exercise and holds great potential, particularly in the field of central nervous system diseases. The research on “exercise mimetics” is based on understanding the mechanisms by which exercise promotes health. Exercise, as a mild stressor, promotes adaptation, thereby enhancing the antioxidant defense system, improving mitochondrial function, and inducing redox remodeling (Cobley et al., 2015). Researchers have proposed the idea that “exercise is an antioxidant,” suggesting the use of antioxidants to mimic the effects of exercise. However, it has been gradually recognized that the health benefits of exercise are not simply due to antioxidation. Instead, exercise reduces ROS to an appropriate level that maintains signaling activity, thereby activating downstream signaling cascades to exert health benefits. Intervening with antioxidants during exercise can actually inhibit these beneficial effects of exercise (Ristow et al., 2009).

Intense or prolonged exercise can increase the production of reactive oxygen species (ROS), leading to elevated levels of oxidative damage biomarkers in muscles and blood (Powers et al., 2020). The production of ROS depends on the mode, intensity, and duration of exercise, which in turn affects the type of adaptive signaling response to oxidative damage (Powers et al., 2020). At low stress levels, neuroimmune activation represents a beneficial protective response; however, at higher stress levels, excessive activation of the neuroimmune system can damage neural networks (Wang et al., 2022) and reduce cognitive performance. Oxidative stress plays a crucial role in the pathological development of Parkinson’s disease (PD) (Bjorklund et al., 2020). Some studies have indicated that oxidative stress induced by LPS injection is mainly controlled by NADPH oxidase, whose expression is increased in PD (Brown and Neher, 2010). Research has found a close relationship between oxidative stress and neuroinflammation (Chakrabarti and Bisaglia, 2023). However, the molecular details of oxidative stress in brain tissue still require further investigation. Exercise can inhibit systemic oxidative stress through adaptive processes, increasing the levels and activities of antioxidants and oxidative damage repair enzymes such as superoxide dismutase and glutathione peroxidase (Patki and Lau, 2011). Soares et al. (2015) recruited 57 healthy men and conducted a 16-week exercise training program. They found that after the training, the participants had significantly reduced DNA strand breaks and FPG-sensitive sites, decreased lipid peroxidation levels, and increased antioxidant activity. This suggests that appropriate exercise may protect lymphocyte DNA from damage by enhancing antioxidant capacity. The beneficial physiological effects of long-term exercise on the body are closely related to promoting suitable levels of ROS production, increasing the activity of antioxidants and damage repair enzymes, and enhancing antioxidant capacity. Additionally, ROS can exert carcinogenic effects through DNA base modifications. For instance, hydroxyl radicals can attack DNA to form 8-hydroxy-2′-deoxyguanosine (8-OH-dG) (Berniyanti et al., 2020). Although intense exercise can increase DNA damage, moderate aerobic exercise can reduce oxidative DNA damage. Regular exercisers have significantly lower levels of 8-OH-dG compared to sedentary individuals, and after 30 min of exercise, the levels of 8-OH-dG in sedentary participants significantly decreased (Asghar et al., 2007). However, our understanding of the specific effects of exercise on mitochondrial function and oxidative stress, and whether myokines play a role in these processes, is still limited and requires further research.

Currently, many issues require further research and discussion, such as: The Impact of Exercise on Oxidative Stress Levels: Investigate how different types and intensities of exercise affect oxidative stress levels. This can be done by measuring oxidative stress indicators, such as oxidative damage biomarkers and antioxidant capacity, to understand how exercise regulates the production and clearance of oxidative stress within cells. The Regulatory Effect of Exercise on the Antioxidant System: Study how exercise regulates the antioxidant system and explore how exercise enhances the cell’s antioxidant capacity. The Impact of Exercise on the Association Between Oxidative Stress and Diseases: Examine the therapeutic effects of exercise as an intervention for diseases related to oxidative stress, such as cardiovascular diseases, cancer, and neurodegenerative diseases. Explore how exercise alleviates the pathological processes of these diseases by regulating oxidative stress levels. The Interaction Between Exercise and Antioxidant Supplements: Investigate the interaction between exercise and antioxidant supplements, and explore the combined effects of exercise and antioxidant supplements on regulating oxidative stress.

## 5 Conclusion

This study used bibliometric methods to perform a visual analysis of research literature from the past 20 years, showcasing the main research content, hotspots, and developmental evolution in the field. The research on exercise-regulated myokines has gone through a period of slow growth, a phase of rapid increase, and is currently in a phase of fluctuating growth. Keyword emergence and evolution prediction maps indicate that exercise types, irisin, and oxidative stress may be research hotspots in the coming years. Analyzing the current major weaknesses and future research hotspots in exercise-regulated myokines research suggests that researchers should focus on the following aspects in the future: 1) The specific differences and mechanisms of action in how different types of exercise regulate myokines. 2) The molecular biological mechanisms of exercise-regulated irisin and the interaction mediated by irisin between muscle and other organs. 3) The significant role of myokine-mediated oxidative stress in exercise mimetics, with an in-depth exploration of the role and clinical application of myokines for specific diseases in clinical practice, aiming to achieve precision medicine.

### 5.1 Limitation

Innovation and Limitations: The innovation of this study lies in the first-time application of CiteSpace, VOS Viewer, and R-bibliometrix to perform bibliometric and visual analyses of exercise-regulated myokine-related literature records indexed in the Web of Science Core Collection from 2003 to 2023. However, there are certain limitations. This study only collected and processed data from a single database, Web of Science, which may cause some bias in the results. Therefore, the overview of this field has certain limitations and needs further refinement.
